# Identification of genomic regions affecting grain peroxidase activity in bread wheat using genome-wide association study

**DOI:** 10.1186/s12870-021-03299-6

**Published:** 2021-11-10

**Authors:** Zhengfu Zhou, Huiyue Guan, Congcong Liu, Ziwei Zhang, Shenghui Geng, Maomao Qin, Wenxu Li, Xia Shi, Ziju Dai, Zhensheng Lei, Zhengqing Wu, Baoming Tian, Jinna Hou

**Affiliations:** 1 Henan Institute of Crop Molecular Breeding, Postgraduate T & R Base of Zhengzhou University, Henan Academy of Agricultural Sciences, Zhengzhou, 450002 China; 2 Agronomy college, Zhengzhou University, Zhengzhou, 450001 China; 3 National Key Laboratory of Wheat and Maize Crop Science, Henan Agricultural University, Zhengzhou, 450002 China

**Keywords:** Wheat, POD activity, GWAS, Flour color, Wheat quality

## Abstract

**Background:**

Peroxidase (POD) activity plays an important role in flour-based product quality, which is mainly associated with browning and bleaching effects of flour. Here, we performed a genome-wide association study (GWAS) on POD activity using an association population consisted with 207 wheat world-wide collected varieties. Our study also provide basis for the genetic improvement of flour color-based quality in wheat.

**Results:**

Twenty quantitative trait loci (QTLs) were detected associated with POD activity, explaining 5.59–12.67% of phenotypic variation. Superior alleles were positively correlated with POD activity. In addition, two SNPs were successfully developed to KASP (Kompetitive Allele-Specific PCR) markers. Two POD genes, *TraesCS2B02G615700* and *TraesCS2D02G583000*, were aligned near the QTLs flanking genomic regions, but only *TraesCS2D02G583000* displayed significant divergent expression levels (*P* < 0.001) between high and low POD activity varieties in the investigated association population. Therefore, it was deduced to be a candidate gene. The expression level of *TraesCS2D02G583000* was assigned as a phenotype for expression GWAS (eGWAS) to screen regulatory elements. In total, 505 significant SNPs on 20 chromosomes (excluding 4D) were detected, and 9 of them located within 1 Mb interval of *TraesCS2D02G583000*.

**Conclusions:**

To identify genetic loci affecting POD activity in wheat grain, we conducted GWAS on POD activity and the candidate gene *TraesCS2D02G583000* expression. Finally, 20 QTLs were detected for POD activity, whereas two QTLs associated SNPs were converted to KASP markers that could be used for marker-assisted breeding. Both *cis-* and *trans-*acting elements were revealed by eGWAS of *TraesCS2D02G583000* expression. The present study provides genetic loci for improving POD activity across wide genetic backgrounds and largely improved the selection efficiency for breeding in wheat.

**Supplementary Information:**

The online version contains supplementary material available at 10.1186/s12870-021-03299-6.

## Background

Flour color is an important parameter for evaluating the end-use quality of flour products, such as noodles and bread [1]. Multiple factors affect flour color, including yellow pigment content, oxidase activity, wheat-grain color, wet gluten content, water absorption, wheat bran content, and protein content. Yellow pigment content, which completely associated with *Phytoene Synthase 1 (Psy1*) gene, is one of the important factors influencing flour yellowness in wheat. Selection of *Psy1* lose function allele would improve flour color through altering carotenoid content [2–4]. Higher protein content can also affect the color of flour products but mainly through darkening dough color via effects on grain hardness, flour texture, and water absorption during the milling process [5].

In addition, colorless precursor substances in flour could produce colored substances through the enzymatic reaction of oxidases, thus affecting flour color. Typically, wheat grains contain three oxidase types: peroxidase (POD), polyphenol oxidase (PPO), and lipoxygenase (LOX). Of these, heme-containing PODs have a wide range of distribution in higher plant cells [6]. The first protein sequence of POD was determined in 1979 [7], and differences in primary structure have resulted in the plant POD super-family being categorized into class I, II, and III [8]. Class I is a group of intracellular enzymes that are extensively present in plants, bacteria, and yeast. This class includes cytochrome C peroxidase (CCP), a soluble protein in the electron transport chain of mitochondria that prevents damage from toxic peroxides; ascorbate peroxidase (AP), which mainly functions to expend hydrogen peroxide in the chloroplast and cytosol of higher plants [9]; and bacterial catalase-peroxidase, which protects cells under oxidative stress [10]. Class II contains four conserved disulfide bonds and two conserved calcium binding sites. They are usually extracellular fungal peroxidases and are assigned as lignin peroxidase or ligninase because they catalyze lignin depolymerization [11]. Class III includes classic and plant-specific peroxidases secreted into the vacuole and cell wall [12], with tissue-specific functions. Previous studies have shown that PODs are involved in various aspects of plant physiology, such as oxidizing compounds with hydrogen peroxide as an electron acceptor, defending against insect attack, generation and detoxification of active oxygen forms, lignin formation, and cell wall biosynthesis [13–15].

Many wheat-grain tissues contain POD, including the epidermis, seed coat, embryo, and endosperm. As a redux enzyme, POD can oxidize ferulic acid and other major phenolic acids, producing chromophoric groups and brown substances [15–17]. High POD activity may darken flour and subsequently result in undesirable pasta color [17, 18]. Indeed, the brown index of pasta products and POD activity are significantly correlated (*r* = 0.84–0.97) [19, 20]. However, when 3000 U POD and 815–1630 U LOX were added to 100 g flour, the dough was completely bleached, indicating that POD could replace benzoyl peroxide as a bleaching agent. Furthermore, POD causes less damage to the flavor of pasta products than LOX when used as the main food additive [21, 22].

Genotype is the primary influencer of POD activity [23–25], although environmental factors also play a role [26] . Previous studies have revealed that genetic variance in POD activity accounts for 90% of total variance [27]. Common wheat has significantly higher POD activity than durum wheat (*P* < 0.05) [28]. Among common wheat varieties, POD activity is fairly variable, differing by 3–10 times [29]. These results suggest that POD activity could be altered through manipulating desired alleles by gene pyramiding. Common wheat contains three subgenomes (A, B, and D), and genes are usually triplicated. Therefore, many copies of POD genes are located on the corresponding homologous regions, distributed throughout wheat A, B, and D genomes [30]. Using CS-nulli-tetrasomic lines, researchers reported POD genes on chromosomes 2A, 2B, 2D, 4B, 7A, and 7D [31]. Quantitative trait loci (QTL) analysis on POD activity using a RIL population (204 lines derived from crossing Doumai and Shi4185) revealed three significant QTLs, *QPod.caas-3AL*, *QPod.caas-4BS*, and *QPod.caas-5AS*, explaining 5.3 ~ 21.2% of phenotypic variation. Functional markers have been developed from the genomic DNA sequence of wheat POD genes [32]. In addition, research that used Gramineae collinearity identified QTL for POD activity through POD genes in rice—specifically, relevant wheat QTL were located on the homologous region of chromosomes 3 and 7, with the former being particularly important [33, 34].

Numerous studies have investigated how LOX and PPO activity are linked to flour color [22], but few have examined the genetic basis of POD activity in wheat association population. To better understand POD genetic diversity in wheat varieties, we conducted a genome-wide association study (GWAS) to detect QTLs associated to grain POD activity in a panel of 207 wheat varieties, and predicted candidate genes. In addition, we developed KASP molecular markers from SNPs located in major loci and evaluated their correlation with POD activity. Our results will greatly contribute to the improvement of flour color and provide theoretical support for advancing wheat molecular breeding.

## Results

### Phenotypic variation in the association population

**Fig. 1:**
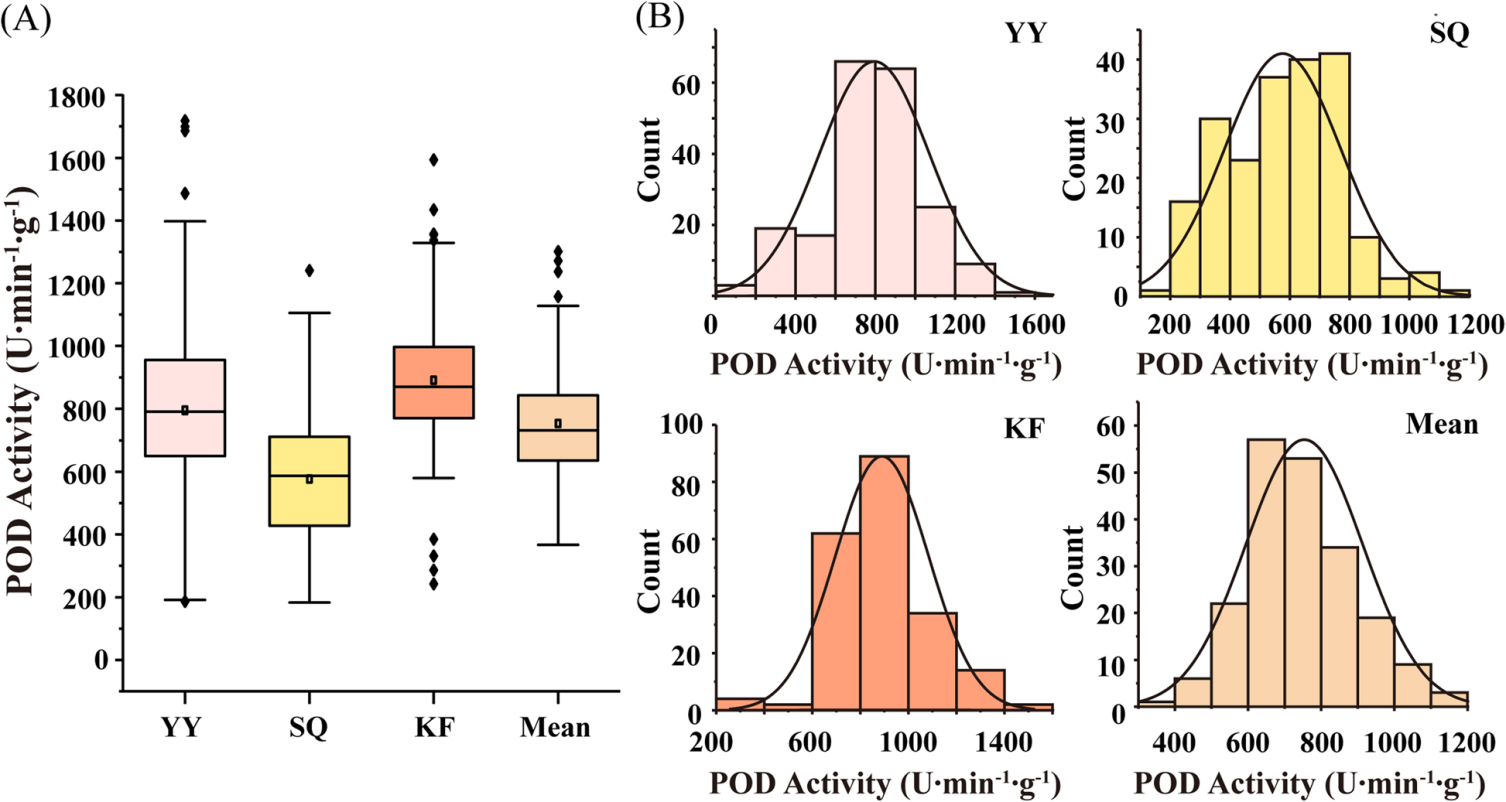
Distribution of POD activity in the association population under different environments. **a** Boxplot of POD activity in Yuanyang (YY), Shangqiu (SQ), and Kaifeng (KF), plus the mean of all three environments. **b** Distribution plots of POD activity in the three environments and their mean

Peroxidase activity was investigated in an association population of 207 varieties planted in three locations. Generally, mean POD activity in the three environments was 753.75 U∙min^**−** 1^∙g^-1**.**^ The highest average POD activity was 889.99 U∙min^**−** 1^**∙**g^**−** 1^ in Kaifeng (KF), and the lowest average was 575.94 U∙min^**−** 1^∙g^**-**1^ in Shangqiu (SQ) (Fig. 1a, Table 1). Maximum POD activity was 1718.42 U∙min^**−** 1^∙g^-1^ in Yuanyang (YY), where the range was 186.00 ~ 1718.42 U∙min^**−** 1^∙g^**-**1^ (Table 1). Minimum POD activity was 182.75 U·min^**−** 1^·g^− 1^ in Shangqiu (SQ), where the range was 182.75 ~ 1240.97 U∙min^**−** 1^∙g^**−** 1^. In all three environments, POD activity exhibited continuous and wide variation that was close to a normal distribution (Fig. 1b). The coefficient of phenotypic variation ranged from 21.84 to 34.67% (Table 1). Estimated heritability for POD activity was 0.51 (Table S2).

**Table 1:** Statistical analyses of POD activity in the association population

Environment^**a**^	Mean ± SD^**b**^ (U∙min^**− 1**^∙g^**-1)**^	Range	Kurtosis	Skewness	CV^**c**^ (%)
YY	795.34 ± 275.73	186.00 ~ 1718.42	1.04	0.24	34.67
SQ	575.94 ± 194.84	182.75 ~ 1240.97	0.01	0.25	33.83
KF	889.99 ± 194.35	242.72 ~ 1593.75	1.53	0.30	21.84
Mean	753.75 ± 160.00	366.93 ~ 1301.30	0.75	0.70	21.23

^a^ Environment: YY (Yuanyang), SQ (Shangqiu), KF (Kaifeng);

^b^ *SD* Standard deviation

^c^ *CV* Coefficient of variation

### QTLs of POD activity identified via GWAS

The QTLs associated with POD activities were detected through GWAS. A total of 20 QTLs with high reliability (−log_10_ *P* ≥ 3 in GWAS and *P* < 0.05 in t-test) were detected associated with POD activity (Table 2). The QTLs were distributed on chromosomes 1D, 2A, 2B, 2D, 3A, 3B, 5A, 6B, 7A, and 7B; they explained 5.59 %~ 12.67% of the phenotypic variation (PVE) (Table 2).

**Table 2:** Information for QTL associated with POD activity identified via GWAS

Num^a^	QTL	Peak SNP	Chr^b^	Position (bp)	Environment^c^	-log_10_***P***	R^2^ (%)
1	*qPOD1D.1*	AX-109976378	1DL	462,948,362	SQ, KF	3.05 ~ 3.24	7.10 ~ 7.26
2	*qPOD2A.1*	AX-108731680	2AS	89,491,698	SQ, KF, BLUP	3.01 ~ 4.47	7.00 ~ 10.16
3	*qPOD2A.2*	AX-111561344	2AL	711,975,486	YY, BLUP	3.33 ~ 4.19	7.64 ~ 9.77
4	*qPOD2A.3*	AX-111137980	2AL	752,916,492	KF, BLUP	3.07 ~ 3.68	5.59 ~ 7.19
5	*qPOD2A.4*	AX-94872128	2AL	774,855,749	SQ, BLUP	3.18 ~ 3.33	6.07 ~ 7.38
6	*qPOD2B.1*	AX-108880049	2BL	712,656,410	YY, BLUP	3.08 ~ 3.70	7.10 ~ 8.48
7	*qPOD2B.2*	AX-110482619	2BL	790,626,375	SQ, BLUP	3.06 ~ 3.43	6.26 ~ 6.96
8	*qPOD2D.1*	AX-111838051	2DL	528,599,809	SQ, BLUP	3.28 ~ 4.08	7.49 ~ 9.23
9	*qPOD2D.2*	AX-108792169	2DL	571,937,591	KF, BLUP	3.89 ~ 4.22	9.12 ~ 9.73
10	*qPOD2D.3*	AX-111564790	2DL	613,093,026	YY, BLUP	3.05 ~ 4.59	5.62 ~ 12.67
11	*qPOD2D.4*	AX-94769224	2DL	648,288,487	SQ, BLUP	3.08 ~ 3.17	6.97 ~ 7.30
12	*qPOD2D.5*	AX-110105841	2DL	648,354,189	SQ, BLUP	3.12 ~ 3.63	6.70 ~ 7.96
13	*qPOD3A.1*	AX-109586344	3AS	11,834,418	KF, BLUP	3.28 ~ 4.23	7.52 ~ 10.12
14	*qPOD3B.1*	AX-95000091	3BS	59,463,829	YY, BLUP	3.12 ~ 3.45	7.11 ~ 7.92
15	*qPOD3B.2*	AX-111774576	3BS	60,381,223	SQ, KF	3.41 ~ 3.50	7.71 ~ 8.23
16	*qPOD5A.1*	AX-109025328	5AS	16,976,687	SQ, KF	3.58 ~ 3.65	8.10 ~ 8.55
17	*qPOD6B.1*	AX-109420494	6BS	227,564,600	SQ, KF	3.09 ~ 3.21	6.90 ~ 7.47
18	*qPOD7A.1*	AX-111699555	7AL	732,587,172	KF, BLUP	3.17 ~ 3.19	7.29 ~ 7.44
19	*qPOD7A.2*	AX-109890531	7AL	736,424,469	SQ, BLUP	3.11 ~ 3.51	7.10 ~ 7.92
20	*qPOD7B.1*	AX-89436260	7BL	742,059,238	KF, BLUP	3.72 ~ 3.77	7.01 ~ 7.04

^a^*Num* Number

^b^*Chr* Chromosome

^c^Environment: YY (Yuanyang), SQ (Shangqiu), KF (Kaifeng), BLUP (Best linear unbiased predictor)

Five QTL were distributed on Chromosome 2DL, concentrated in the genomic region of 528.60–648.35 Mb, which anchors the end of the chromosome close to the telomere. Interestingly, three QTL detected on chromosome 2AL were also located in the genomic region near the telomere.

**Fig. 2:**
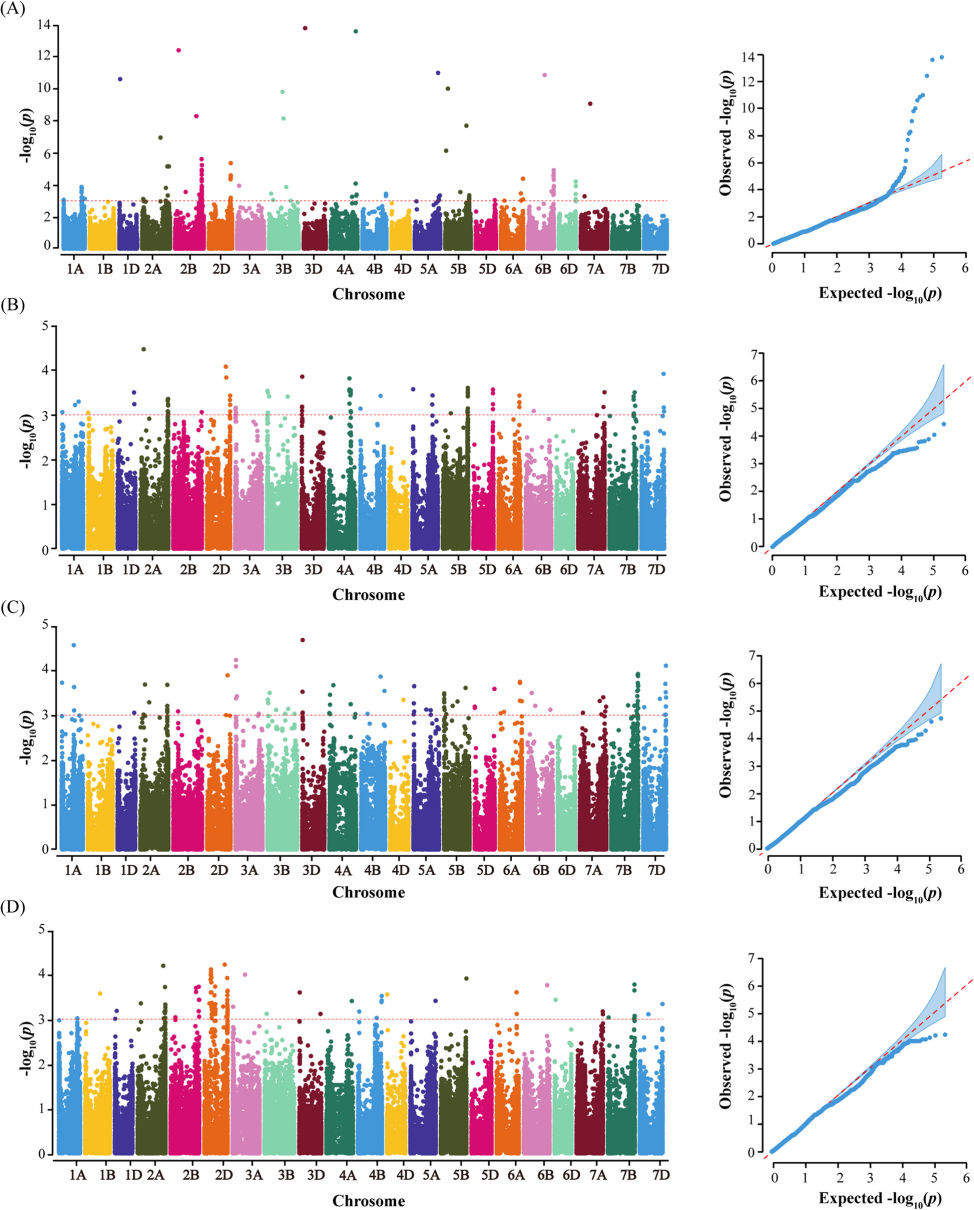
Manhattan and *Q-Q* plots for POD activity in 207 wheat varieties. **a**, **b**, **c**, and **d** represent Manhattan and *Q-Q* plots based on GWAS of POD activity across Yuanyang, Shangqiu, Kaifeng and the BLUP values of the three environments, respectively

We mined the fewest (four) QTL from YuanYang, distributed on chromosomes 2A, 2B, 2D, and 3B, with a PVE range of 5.62–12.67%. However, *qPOD2D.3* on chromosome 2D (613.09 Mb), contributed the most to PVE (5.62–12.67%) in this environment. In SQ, we detected 11 QTLs distributed on chromosomes 1D, 2A, 2B, 2D, 3B, 5A, 6B, and 7A, with a PVE of 6.07–10.16%. In KF, 10 QTLs were distributed on chromosomes 1D, 2A, 2D, 3A, 3B, 5A, 5B, 7A, and 7B (Fig. 2, Table 2), contributing 6.90–10.16% to phenotypic variation. Among all identified QTL, 16 were found using combined data across the three environments (best linear unbiased predictor, BLUP), while four QTL (*qPOD1D.1*, *qPOD3B.2*, *qPOD5A.1,* and *qPOD6B.1*) were found only in SQ and KF. One QTL (*qPOD2A.1*) on chromosome 2A (89.49 Mb) was repeatedly detected in three environments, SQ, KF, and BULP (Table 2). Fifteen QTL (four in YY, six in SQ, and five in KF) were screened only in one specific environment and BLUP.

### Evaluation of superior and inferior alleles

For each SNP anchored to the peak of the QTL, the allele contributing to increase POD activity was designated as the superior allele, while the allele that decreased POD activity was inferior. Significant SNP AX-95000091 had the most heterozygous genotypes, at 165. Five significant SNPs (AX-111137980, AX-94872128, AX-110482619, AX-110105841, and AX-89436260) had two homozygous genotypes and no heterozygous genotypes were detected in the present association population (Table 3).

**Table 3:** Analysis of variance for individuals harboring different genotypes for the 20 SNPs

Num	SNP	chr	Genotype^**1**^			No. of varieties ^**2**^			POD activity(U∙min^**−1**^∙g^**−1**^)		
1	AX-109976378	1D	CC	TT	TC	164	42	1	740.49b	793.23a	–
2	AX-108731680	2A	CC	TT	TC	178	4	25	738.47c	1070.34a	811.95b
3	AX-111561344	2A	TT	CC	CT	81	121	2	718.41b	772.48a	–
4	AX-111137980	2A	TT	CC	None	64	143	None	724.45b	766.87a	None
5	AX-94872128	2A	TT	CC	–	18	188	–	637.27b	765.95a	–
6	AX-108880049	2B	GG	AA	AG	56	4	146	733.18b	1122.97a	751.13b
7	AX-110482619	2B	CC	TT	None	20	187	None	637.79b	766.16a	None
8	AX-111838051	2D	CC	GG	GC	80	125	2	678.68b	798.17a	–
9	AX-108792169	2D	GG	CC	CG	158	8	41	760.04b	1062.85a	669.23c
10	AX-111564790	2D	CC	TT	TC	137	10	60	759.75b	942.44a	708.62c
11	AX-94769224	2D	TT	CC	CT	19	136	48	633.08c	781.65a	722.47b
12	AX-110105841	2D	CC	GG	None	19	188	None	633.08b	765.95a	None
13	AX-109586344	3A	TT	GG	GT	127	78	1	726.1b	789.04a	–
14	AX-95000091	3B	GG	CC	CG	37	5	165	690.65c	1112.33a	757.04b
15	AX-111774576	3B	AA	GG	GA	134	3	66	753.94b	1104.68a	731.77b
16	AX-109025328	5A	GG	AA	AG	139	66	1	731.06b	791.12a	–
17	AX-109420494	6B	TT	GG	GT	109	97	1	729.74b	775.41a	–
18	AX-111699555	7A	GG	TT	TG	43	92	70	720.42b	793.51a	722.94b
19	AX-109890531	7A	GG	AA	AG	89	12	104	698.84c	744.14b	803.69a
20	AX-89436260	7B	GG	AA	None	113	94	None	721.98b	791.95a	None

The values in the column “No. of varieties” and “POD activity” were in turn corresponding to the genotypes in column “Genotype”. “None” indicated no such values were detected in association population. “**–**” represented the uncertainty or missing values. The lowercase letters a, b, and c indicate significant differences after an analysis of variance (*P* < 0.05)

^1^ Genotypes detected from 20 SNPs significantly associated with POD activity. The two letters represented the SNPs from two alleles, respectively

^2^The total variety number may not add up to 207 in cases the genotype of some varieties were missing

Six SNPs (AX-108731680, AX-108792169, AX-111564790, AX-94769224, AX-95000091, and AX-109890531) showed additive effects on POD activity. Three SNPs (AX-108880049, AX-111774576, and AX-111699555) had dominant effect on POD activity (*P* < 0.05). We could not conduct multiple comparisons on six SNPs (AX-109976378, AX-111561344, AX-111838051, AX-109586344, AX-109025328, and AX-109420494), because they had less than two heterozygous genotypes.

**Fig. 3:**
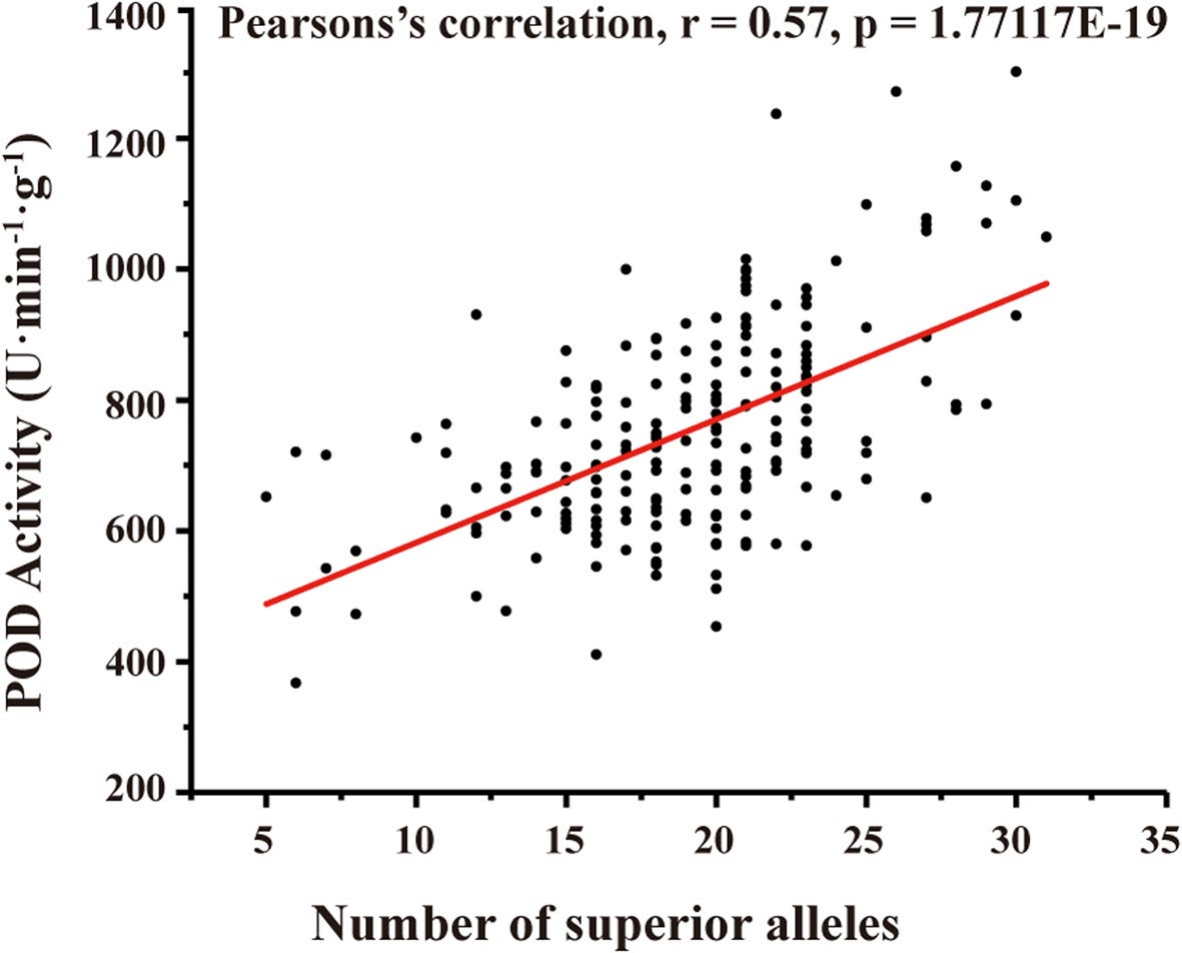
Correlation analysis between number of superior SNP alleles and POD activity

Aggregation analysis showed that POD activity was positively correlated with number of superior alleles in the 207 wheat varieties (*r* = 0.57, Fig. 3). The more superior alleles a variety contained, the higher its POD activity in the grain. For instance, the variety Shannong26 which containing the highest number of superior alleles (31 alleles) displayed the highest POD activity of 1301.30 U∙min^− 1^∙g^− 1^. Similarly, the varieties such as Jimai22 and Yuanzhu which contained 30 superior alleles, showed relatively higher POD activity of > 1000 U∙min^− 1^∙g^− 1^ (Table S4).

To benefit genotyping and selection for breeding, we developed two SNPs as KASP markers. One of the KASP was developed from the SNP AX-109420494 which located under the peak of the major QTL - *qPOD6B.1* (Table S5). However, for another major QTL, *qPOD2A.1*, it was failed to develop KASP marker from the SNP AX-108731680 under the peak of the QTL. Therefore, the SNP AX-111134186 which distancing only 0.0056 Mb from the SNP AX-108731680 was selected and successfully developed for KASP marker. Efficiency of the two KASP markers was certified using mean POD activity across the three environments and BLUP. Superior alleles of AX-111134186 and AX-109420494 increased POD activity by 11% and 6% compared with inferior alleles, respectively (Table S6).

### Prediction of candidate genes for POD activity in the association population

**Fig. 4:**
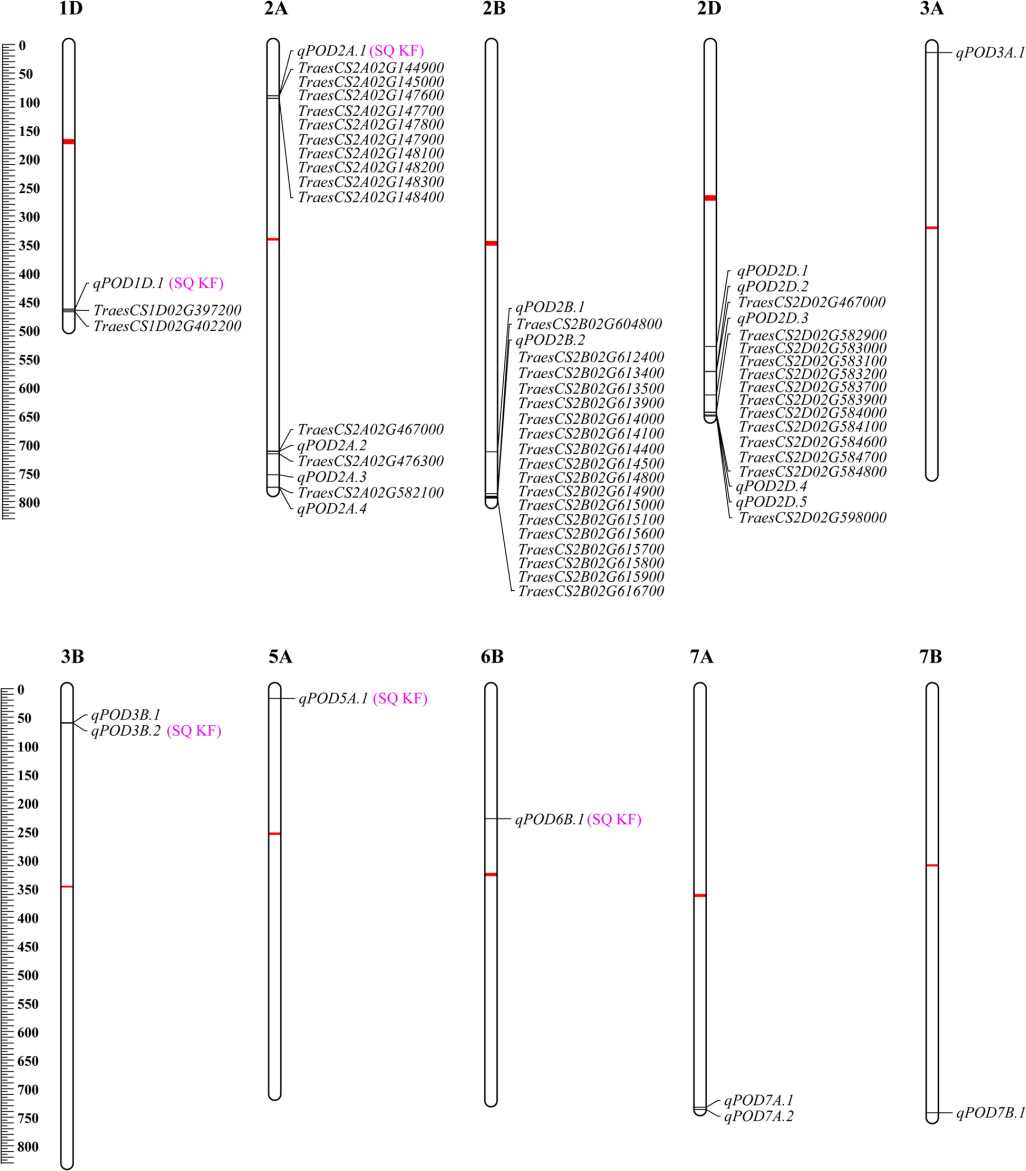
Physical maps of the 46 POD genes within 10 M interval of QTL identified by GWAS of POD. The centromere structure was marked by red rectangles. Among 20 QTL, five were association with POD activity in two environments, Shangqiu (SQ) and Kaifeng (KF)

**Fig. 5:**
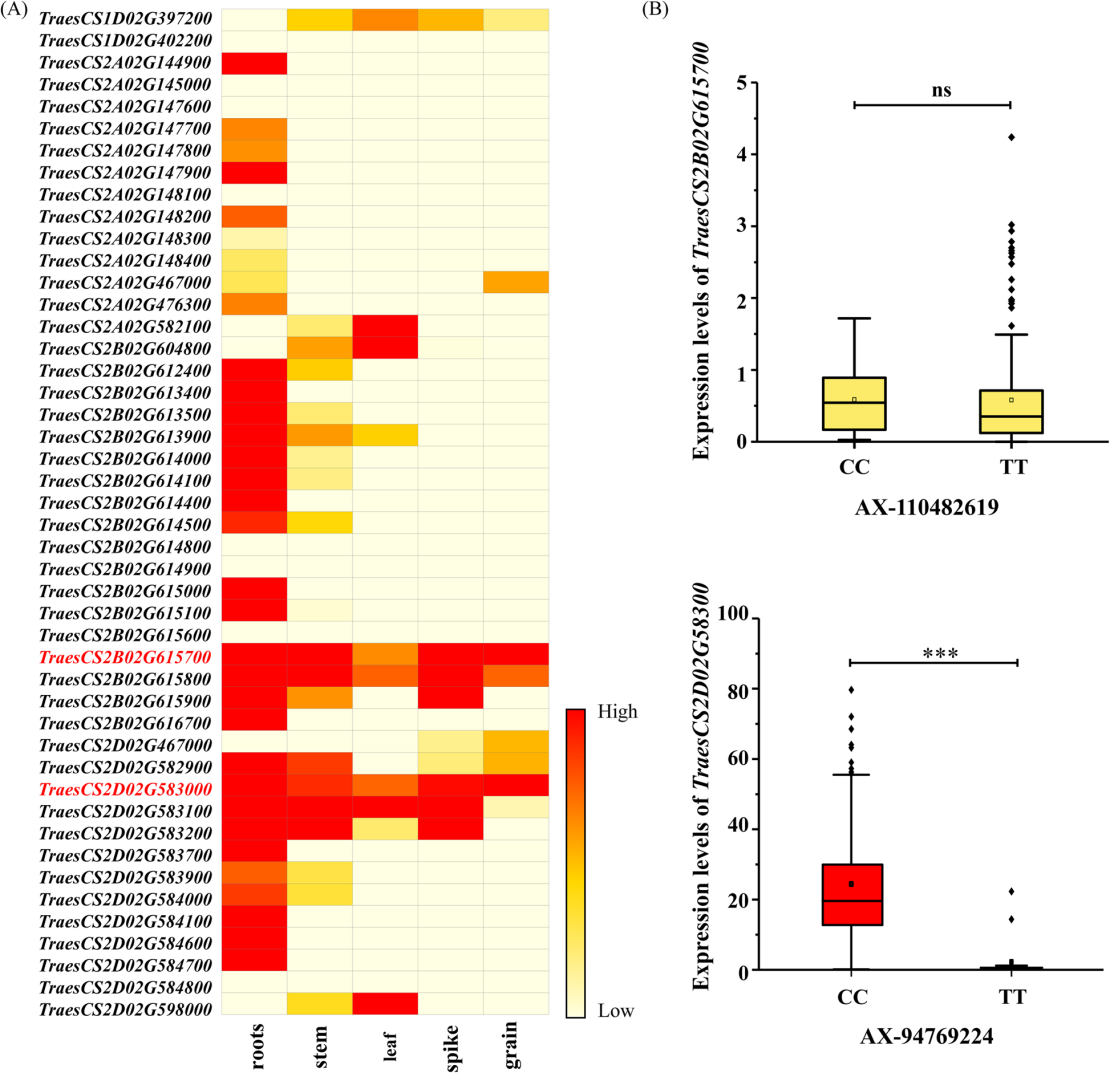
Prediction of candidate genes for POD activity. **a** The expression levels of POD genes within 10 M interval of QTL identified by GWAS of POD in different wheat tissues. **b** The expression levels of *TraesCS2B02G615700* and *TraesCS2D02G583000* between the cultivars with SNPs AX-110482619 and AX-94769224. ***:*P* < 0.001; ns: no significant

We screened 46 POD-encoding genes within 10 Mb of the sequence flanking QTL (5 Mb upstream and downstream), based on genome annotations of ‘Chinese Spring’ by the International Wheat Genome Sequencing Consortium (IWGSC) and the Wheat Expression Browser (Fig. 4 and Table S7). Interestingly, most genes were highly expressed in roots, while only two genes were highly expressed in grain, these two were *TraesCS2B02G615700* (2B:793.46–793.46 Mb) and *TraesCS2D02G583000* (2D:643.44–643.44 Mb), targeting QTL *qPOD2B.2* and *qPOD2D.4*, accordingly (Fig. 5a).

The SNPs AX-110482619 (C/T) and AX-94769224 (T/C) associated with QTLs *qPOD2B.2* and *qPOD2D.4*, accordingly, were used for genotyping the 207 tested varieties. Meanwhile, the expression level of *TraesCS2B02G615700* and *TraesCS2D02G583000*, which anchored the corresponding QTL, were detected at 20 days after pollination (DAP) in grains (Table S8). There was no expression divergence of *TraesCS2B02G615700* between cultivars with genotype CC and TT in the SNP AX-110482619 locus. Nevertheless, the expression level of *TraesCS2D02G583000* between cultivars with genotype TT and CC in the locus AX-94769224 (Fig. 5b, Table S8) were significantly diverged (*P* < 0.001).

**Fig. 6:**
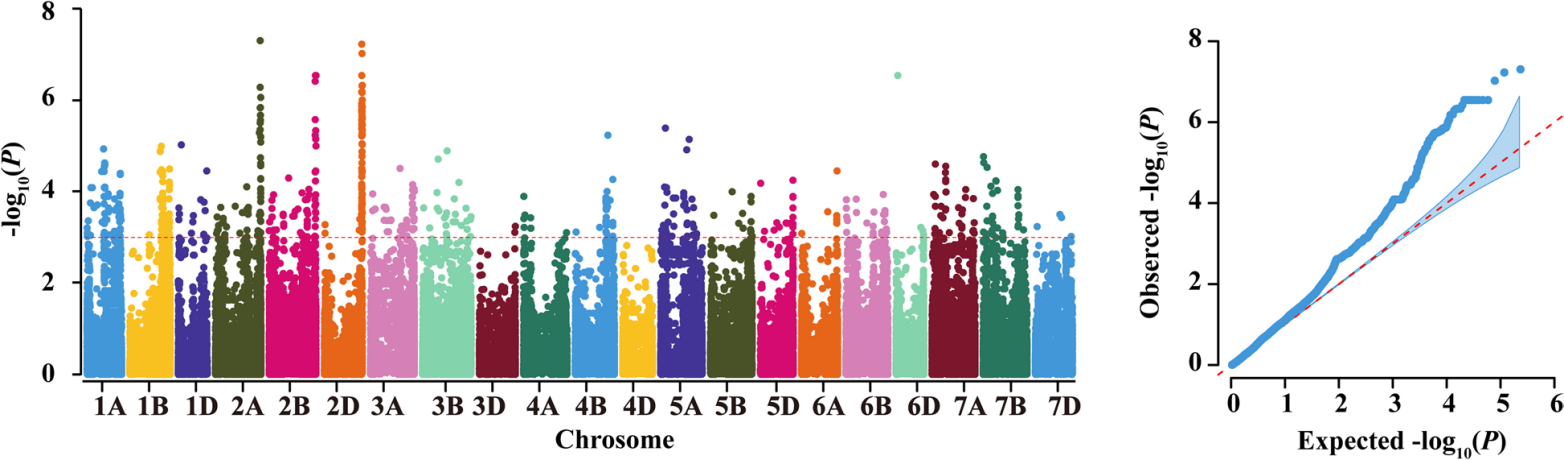
The Manhattan plots and *Q*–*Q* plots of the expression levels of *TraesCS2D02G583000*

*TraesCS2D02G583000* expression was used to conduct expression GWAS (eGWAS) and determine whether regulatory element exists in other genetic loci (Fig. 6). Five hundred and five significant SNPs on 20 chromosomes (excluding 4D) in the wheat genome by eGWAS. The PVE ranged from 5.44% to 15.61% (Table S9). The significant SNPs screened by eGWAS distributed unevenly on chromosomes. Chromosome 2D concentrated the most number of significant SNPs which the total number was 56 and there were 2 and 54 distributed on the short and long arms, respectively. These SNPs explained 6.66–15.55% of phenotypic variation. Chromosome 3D contained the fewest number (2) of significant eGWAS SNPs. The homelogous group 2 chromosomes (2A, 2B and 2D) were recognized the most important region revealed by eGWAS. The significant SNPs located on homelogous group 2 chromosomes showed relatively higher -log_10_*P* value (3-7.3) than that in other chromosomes and accounted for the highest contribution to the phenotypic variation (5.75–15.61%) (Table S9). Four SNPs (AX-94872128, AX-110482619, AX-94769224, and AX-110105841) were significantly associated with both POD activity and *TraesCS2D02G583000* expression. Nine significant SNPs were within 1 Mb of *TraesCS2D02G583000*, with AX-108833042 being only 1536 bp away*.*

## Discussion

In this study, we used a population consisting of 207 wheat varieties to perform a GWAS on POD activity at three locations. We detected 20 QTLs that were significantly associated with POD activity chromosomes 1D, 2A, 2B, 2D, 3A, 3B, 5A, 6B, 7A, and 7B. Previous studies have also identified three of the QTLs (*qPOD2A.4*, *qPOD2D.4*, and *qPOD2D.5*), located on chromosomes 2A and 2D [35]. In addition, two QTLs on 2D (*qPOD2D.4* and *qPOD2D.5*) were similar to those detected in Gaocheng8901/Zhoumai16 recombinant inbred lines (RILs) by using 90 K SNP chips [1]. These results indicated a universal function of the locus in 2A and 2D and suggest that the identified SNPs may be part of key loci for POD activity. The panel we used consisted of varieties originating from various planting areas worldwide, with a wide genetic background and variation than populations used in previous studies, which had only Chinese varieties. Thus, we found 17 QTLs associated with POD activity. Our results provide a locus that is more functional for improving POD activity across wide genetic backgrounds and may enhance breeding efficiency.

We then identified 46 POD genes in 20 QTL flanking regions based on the Chinese Spring genome in Ensemble plant database (http://plants.ensembl.org/index.html). Genes related to POD have various biological functions, such as biotic and abiotic stress resistance, lignification, and seed development; they have also been linked to the rheological properties of flour dough. Due to these wide-ranging functions, the expression patterns of different POD genes are divergent in wheat, exhibiting tissue- and developmental stage- specificity. Some genes were highly expressed in all tissues, including coleoptile, root, and embryo in the germinating seed stage; root, crown, and leaf in the seedling stage; and immature inflorescences. Other genes displayed low expression levels. Previous reports have described that *POX1*, *POX2,* and *POX4* encoded POD were primarily expressed in roots. In our present study, most of the POD genes within 10 Mb of the QTLs were highly expressed in the roots, which was consistent with previous studies [36]. Moreover, two POD genes, *TraesCS2B02G615700* and *TraesCS2D02G583000*, were detected highly expressed in wheat grains. Therefore, we prioritized those SNPs on chromosomes 2B and 2D for functional marker development, which should benefit molecular marker-assisted breeding in wheat. Our results revealed that SNPs significantly influence POD activity in the association population, potentially contributing to ubiquitous POD activity in wheat. The eGWAS detected multiple loci, distributed in nearly every chromosome, that influence *TraesCS2D02G583000* expression, with of which some were close to the candidate gene. Therefore, we speculate that both *cis-* and *trans-* acting elements may regulate *TraesCS2D02G583000* expression. Although our findings provide clues for the molecular mechanisms underlying POD gene regulation, this topic requires further investigation.

Although POD activity is used as a physical and chemical indicator, the detection procedure is cumbersome and time-consuming, given difficulties in measuring all individuals and selecting elite lines from the early generation of breeding populations. Developing and utilizing functional markers is an efficient and economical approach for large-scale screening of high POD activity lines and individuals. Furthermore, based on polymorphisms of functional genes, molecular-assisted breeding has been widely used for wheat [37–39]. However, no molecular markers are available for POD gene selection in breeding, restricting the genetic improvement of wheat POD activity. Based on two QTL (AX-111134186 and AX-109420494) associated with POD activity on chromosomes 2A and 6B, we developed KASP markers and certified their efficiency in an association population. Further research should yield more KASP markers targeting SNPs associated with POD activity. Combining multiple major genetic sites can significantly improve the efficiency of molecular marker-assisted selection breeding for high POD activity in wheat.

## Materials and methods

### Phenotypic variation in the association population

The population used for GWAS comprised 207 wheat cultivars, which includes typical cultivars from Yellow - Huai Valleys and the Southwestern Wheat Region of China collected by the Quality Breeding Group of Wheat Research Institute, Henan Academy of Agricultural Sciences from Henan Province Crop Germplasm Bank, as well as 7 other countries provided by The International Maize and Wheat Improvement Center (CIMMYT) (Table S1). The authors declare the total permissions to use the collections. Collected cultivars represent a wide range of genetic resources in the world. The population was planted in Yuanyang (E113°97′, N35°5′), Shangqiu (SQ, E115°65′, N34°45′), and Kaifeng (E114°30′, N34°80′) of Henan Province, China, during the 2016–2017 planting season. All cultivars were planted in two rows of 2 m length, separated by 10 cm in width and arranged in a randomized complete block. Field management was organized according to local practices at each site.

### Phenotypic investigation and data collection

For each sample, 5 μL of POD whole-wheat extract was added to 175 μL of a substrate mixture comprising 25 μL of 2% hydrogen peroxide, 5 μL of 2% guaiacol, and 145 μL citrate phosphate buffer (pH = 5.0) [32]. Guaiacol in the mixture was catalyzed via the reduction of hydrogen PODs, taking electrons from hydrogen peroxide and producing a dark-brown product with maximum absorbance at 470 nm. To quantify POD activity, change in absorbance at 470 nm was measured using a UV-1800PC spectrophotometer (MAPADA). Each sample had two replicates, and their means were used as the phenotype for analysis.

### Statistical analysis of phenotypic data

Descriptive statistics of phenotypic data (POD activity) from each location (Yuanyang, Shangqiu and Kaifeng) was conducted in R version 3.5.1 with the “psych” package. Correlations and variance of phenotypes were analyzed in IBM SPSS Statistics. Broad-sense heritability (*H*^*2*^) was calculated in R package lme4, according to the formula *H*^*2*^ = ơ^2^g/(ơ^2^g + ơ^2^ge/e + ơ^2^ε/er) (ơ^2^g represents genetic variance; ơ^2^ge indicates variance of genetic-environmental interaction; ơ^2^ε indicates residual variance; e is number of environments; r is number of replicates in a single environment). Best linear unbiased predictor (BLUP) values were calculated using the formula Y = (1|Line) + (1|Loc) + (1|Line: Loc) where “Y”, “1|”, “Line”, and “Loc” represent trait value, groups, all testcrosses, and environments, respectively. These BLU*P* values for POD activity were used as phenotypic data from an independent environment for further analysis.

### Genome-wide association analysis

Genome-wide association analysis was performed on wheat-grain POD activity in the wheat grain of association population in three environments and BLUP by using the mixed linear model (MLM) [40]. Population structure and kinship based on 224,706 high-quality SNP markers which developed from the Wheat Breeders 660 K Axiom® array and POD activity of 207 wheat varieties were imported into TASSEL version 5.0 [41, 42]. A threshold of *P* value at 0.001 was used to determine significant marker-trait associations. The false discovery rate (FDR) was calculated using SAS to determine the significance level of the SNP with *P*-value < 0.05 (Table S10) [43, 44]. All the SNPs were calculated pair-wise linkage disequilibrium (LD) (Fig. S1) as their squared correlation coefficients (*r*^*2*^) and LD decay of the whole genome in the association population was evaluated using TASSEL version 5.0. The LD decay was assigned as the corresponding distance at half of maximum *r*^*2*^ value. Every stable, significant SNP for POD activity was above the threshold and was associated with more than two environments. Manhattan plots and *Q*–*Q* (Quantile–quantile) plots were created using the CMplot code (https://github.com/YinLiLin/R-CMplot) in R version 3.5.1 [45].

### KASP marker development and genotyping

PolyMarker (http://www.polymarker.info/) was used to design KASP primers. The PCR reaction assay contained primer mix, KASP master Mix and DNA and amplification was performed using the CFX96 Touch™ real-time PCR detection system. The thermocycling schedule was as follows: initial denaturation at 95 °C for 15 min, 10 touchdown cycles (95 °C for 20 s; touchdown at 65 °C initially and decreasing by 1 °C per cycle for 25 s), 30 additional cycles (95 °C for 10 s; 57 °C for 60 s), and three extension steps (each cycle of annealing: 95 °C for 10 s, 57 °C for 60 s) [46]. At the end of PCR, BIO-RAD automatically determined sample genotype based on position in the allelic discrimination plot. The value of POD activity with different genotypes were calculated according to the method described previously [47].

### Candidate gene prediction for POD activity

Two approaches were used to predict candidate genes for POD activity in associated genomic regions. The first approach referred to gene annotations of the ‘Chinese Spring’ functional gene database IWGSC (RefSeq version 1.1). All high confidence POD genes within 10 Mb physical intervals from the QTL were used for screening candidate genes. The second approach used gene expression profiling from the Wheat Expression Browser (http://www.wheat-expression.com); highly expressed genes in the grain were chosen as candidate genes.

### Genome-wide association study of POD gene expression

Grains of 207 cultivars in the association population were used for RNA sequencing at 20 DAP, as previously reported [41]. Peroxidase-gene expression levels (represented as FPKM) were also used as a phenotype for GWAS with a consistent statistics model and threshold. *Cis*-acting elements were thought to be regulators if a significant SNP site was scanned within 1 Mb interval of the corresponding gene; otherwise, trans-regulation was considered the primary influencer.

## Supplementary Information

**Additional file 1 **: **Table S1.** The POD activity (U·min^− 1^·g^− 1^) in 207 wheat accessions of each environment and BLUP. **Table S2.** Analysis of variance (ANOVA) and broad-sense heritability for POD activity based on three environments. **Table S3.** Marker-trait associations for POD activity in the associated population analyzed by the mixed linear model (MLM). **Table S4.** Number of superior alleles across 20 significantly associated SNPs in the genome of 207 wheat varieties. **Table S5.** Primers designed based on KASP markers AX-109420494 and AX-111134186. **Table S6.** The relationship between superior and inferior alleles of KASP markers to POD activity. **Table S7.** The expression values of high-confidence POD genes within 10 Mb physical intervals from the significant SNPs. **Table S8.** The expression of *TraesCS2D02G583000* between the varieties in association population based on the genotypes AX-94769224 and AX-110482619. **Table S9.** The significant SNPs of GWAS analysis conducted by the expression levels of *TraesCS2D02G583000*. **Table S10.** The false discovery rate of the 20 significant SNPs.

**Additional file 2 **: **Figure S1.** Linkage equilibrium decay plots of *r*^*2*^ over physical distance in the association population.

## Data Availability

The datasets necessary for supporting the results of this manuscript are included in this manuscript (and its supplementary files). The RNA-seq data are available in Genome Sequence Archive (https://bigd.big.ac.cn/gsa/browse/CRA004223).

## Abbreviations

POD: Peroxidase
MLM: Mixed liner model
GWAS: Genome-wide association study
BLUP: Best linear unbiased predictor
SNP: Single nucleotide polymorphism
*Q-Q*: Quantile-quantile
PVE: Phenotypic variation explained
IWGSC: International Wheat Genome Sequence Consortium
KASP: Kompetitive Allele-Specific PCR
QTL: Quantitative trait locus
DAP: Days after pollination
