# Supplementary figures and images for: Identification of genomic regions affecting grain peroxidase activity in bread wheat using genome-wide association study
Source: BMC Plant Biol. 2021 Nov 10;21:523. doi: 10.1186/s12870-021-03299-6 (PMC8579651; doi:10.1186/s12870-021-03299-6)

## LD decay

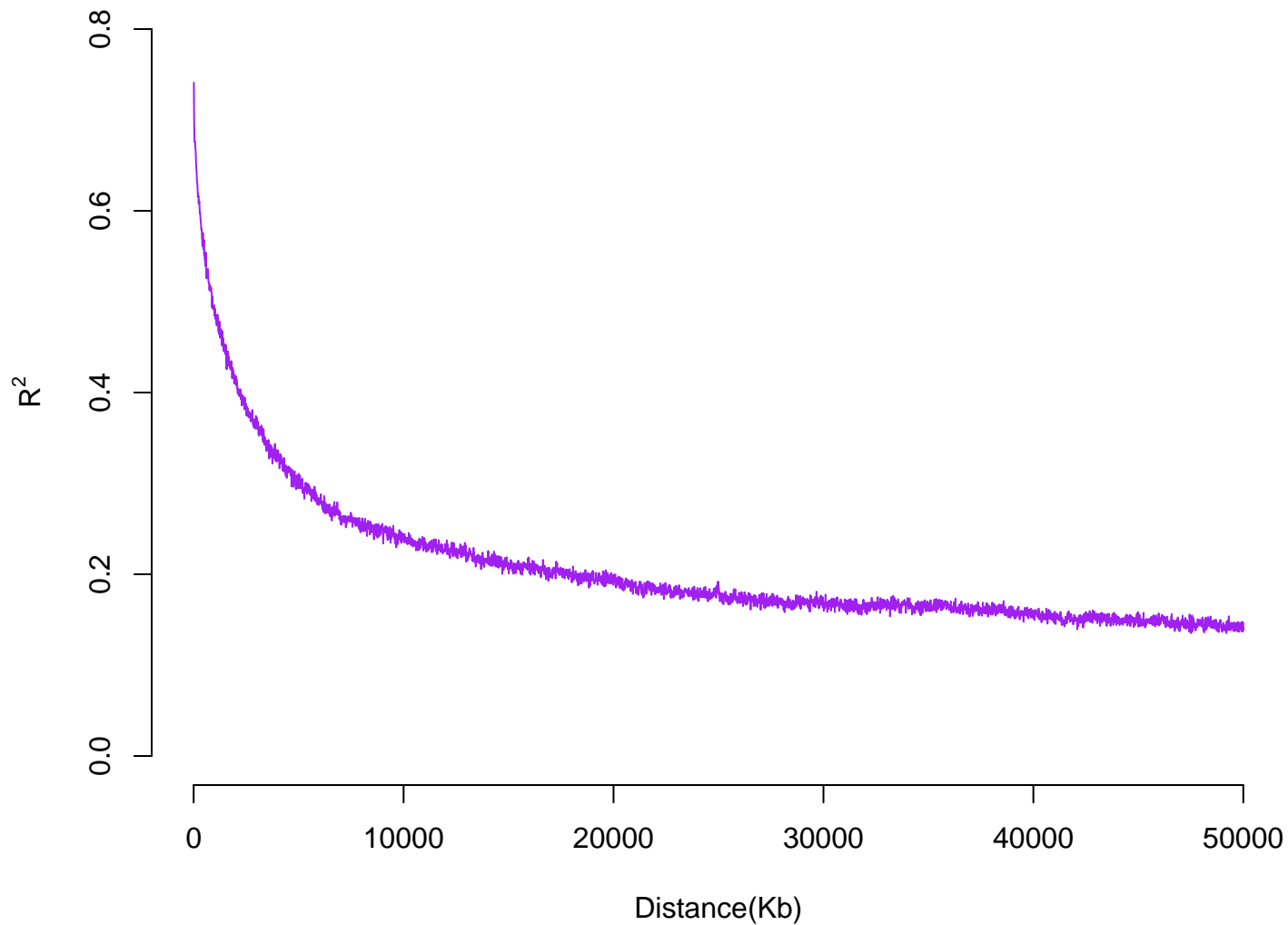

Supplement: Supplementary file 2 — Additional file 2 : Figure S1. Linkage equilibrium decay plots of r2 over physical distance in the association population. [file 12870_2021_3299_MOESM2_ESM.pdf]
